# Multi-Year Analysis of Respiratory Viral Dynamics Reveals Significance of Rhinovirus in Young Children with Severe Respiratory Illness

**DOI:** 10.3390/idr17020029

**Published:** 2025-04-03

**Authors:** Juan Raphael Caldera, Tawny Saleh, Trevon Fuller, Shangxin Yang, Karin Nielsen-Saines

**Affiliations:** 1 Department of Pathology and Laboratory Medicine, David Geffen School of Medicine, University of California, 11633 San Vicente Blvd, Brentwood Annex, Los Angeles, CA 90049, USA; juanraphael.f.caldera@questdiagnostics.com (J.R.C.); shangxinyang@mednet.ucla.edu (S.Y.); 2 Division of Preventive Medicine, David Geffen School of Medicine at UCLA, Los Angeles, CA 90095, USA; 3 Division of Informatics, Department of Computational Biomedicine, Cedars-Sinai Health System, Los Angeles, CA 90048, USA; 4 Division of Pediatric Infectious Diseases, Department of Pediatrics, David Geffen School of Medicine, University of California, 22-442 MDCC Box 951752, Los Angeles, CA 90095, USA; trevonfuller@mednet.ucla.edu (T.F.); knielsen@mednet.ucla.edu (K.N.-S.)

**Keywords:** respiratory virus, rhinovirus, pediatric infections

## Abstract

**Objectives**: We aimed to analyze the landscape of viral respiratory illnesses (VRIs) in a large metropolitan area in Southern California with a focus on the COVID-19 pandemic. **Methods**: We conducted a retrospective cohort study within the UCLA Health System, which evaluated children aged 0–5 years who received comprehensive respiratory viral panel (cRVP) testing during August–February of 2018–2023. The patient demographics, disease severity, and clinical course were specifically compared during the pandemic. Predictors of significant VRI were determined by multivariate logistic regression. **Results**: A total of 1321 children underwent cRVP testing, and 753 positive subjects were identified during the study period. Rhinovirus (RV) was by far the most frequent virus detected across 5 years, even during the COVID-19 pandemic, followed by respiratory syncytial virus (RSV). Along with RSV and human metapneumovirus, RV was identified as an independent risk for significant disease and occurred irrespective of co-infection with other viruses. **Conclusions**: RV was the most common viral pathogen in young children, even during the height of the COVID-19 pandemic, and was an independent driver of moderate-to-severe disease, particularly in children with comorbidities. Ethnic disparities were also observed as a risk for significant disease, underscoring the need for targeted interventions and heightened clinical vigilance in pediatric populations.

## 1. Introduction

On average, children less than 2 years of age acquire 2–8 viral respiratory infections (VRIs) annually, and as many as 14 for those attending daycare [1,2]. A recent community-based longitudinal study in young children revealed that while the majority of infections remain asymptomatic, as many as 40% cause acute respiratory illnesses with significant symptoms, at times leading to secondary bacterial infection or non-judicious use of antibiotics [2]. While VRIs are typically self-limiting, lasting 7–9 days, as many as 13% may last longer and progress to severe manifestations leading to emergency department (ED) visits and hospital admissions [1]. Estimates of the financial cost of hospitalizations due to VRIs approximated to USD 2800–3200 per child, compounding associated medical concerns with significant economic burden [3].

To mitigate the broader impact of VRIs, the CDC employed the Respiratory Virus Hospitalization Surveillance Network (RESP-NET), which comprises three networks (COVID-NET, RSV-NET, and FluSurv-NET) that conduct population-based surveillance of laboratory-confirmed hospitalizations associated with the 2019 severe acute respiratory syndrome coronavirus (SARS-CoV-2), respiratory syncytial virus (RSV), and influenza (flu). The metrics measured by RESP-NET are intended to provide an accessible and up-to-date platform to follow trends in hospitalization due to these three pathogens across various demographics and seasons [4]. Under the same intent, many independent studies also investigated the epidemiology of VRIs to understand viral dynamics within a population and its consequent burden of disease.

Before the COVID-19 pandemic, VRIs followed a typical seasonal pattern, with flu, non-SARS-CoV-2 coronaviruses (CoV), and RSV peaking in the winter [5,6]. Adenovirus, human bocavirus, human metapneumovirus (hMPV), and rhinoviruses (RVs) are detected year-round [5,6]. However, since the COVID-19 pandemic, reports from the US, Australia, Europe, and South America indicated a shift in respiratory virus epidemiology, with reduced numbers during the usual season and an interseason outbreak [7,8,9,10,11]. Non-pharmacological interventions, such as the use of face masks, lockdowns, and travel restrictions implemented to prevent SARS-CoV-2 transmission, contributed to this change, thus leading to global declines in respiratory viruses [12,13,14,15,16,17,18,19,20,21,22,23,24]. Given the accumulating data alluding to the changing landscape of VRIs in the COVID-19 era, we sought to assess respiratory virus prevalence in a healthcare system that provides up to quaternary medical care to a large metropolitan population. The objective of our study was to understand viral dynamics within a susceptible pediatric population of 5 years or younger and identify risks for the development of more severe disease and consequent hospitalization.

## 2. Methods

**Study setting and design:** We conducted a retrospective cohort study at a large academic medical center (Ronald Reagan Medical Center and Mattel Children’s Hospital at UCLA), a community hospital (UCLA Santa Monica Hospital), and a widespread network of UCLA-affiliated clinics throughout Los Angeles County.

**Inclusion criteria:** Children 0–5 years who underwent comprehensive respiratory viral panel (cRVP) testing at our hospital facilities and affiliated clinics across Los Angeles County during visits to acute care pediatric settings and emergency departments or during inpatient admission. **Exclusion Criteria:** Children who did not receive cRVP testing. cRVP includes RSV/flu and the respiratory viral panel (RVP) with RT-qPCR. SARS-CoV-2 RT-qPCR testing was included during the COVID-19 pandemic. Patients were selected based on positive RT-qPCR results. If a participant had multiple tests performed, the first positive result was used for the analysis. For patient analyses, each participant was counted once, even if more than one test was performed during the clinical encounter within the given viral season. The study period encompassed five respiratory viral seasons from August to February in 2018–2019, 2019–2020, 2020–2021, 2021–2022, and 2022–2023, corresponding to the peak time of respiratory virus circulation.

**Respiratory pathogen evaluations:** The GenMark ePlex Respiratory Viral Panel platform (GenMark Diagnostics, Inc., Carlsbad, CA, USA) was used throughout the five consecutive time periods for detection of a panel of respiratory viruses collected by a nasal swab. The platform detects 8 viral pathogens including adenovirus A-F, CoV 229E/HKU1/NL63/OC43, hMPV, human RV/enterovirus, flu A/A H1/A H1-2009/A H3, flu B, parainfluenza 1/2/3/4, and RSV A/B. SARS-CoV-2 qPCR was performed separately using various platforms: DiaSorin Molecular Simplexa^TM^ (DiaSorin Molecular LLC, Cypress, CA, USA), Roche cobas^®^ 6800 (Roche Diagnostics, Indianapolis, IN, USA), or Hologic Panther Aptima (Hologic, Inc., Marlborough, MA, USA). These viruses were selected as they were predominantly identified in our pediatric population. Due to limitations of the testing platforms, enterovirus could not be distinctly excluded from the RV group; however, given the anatomical site of specimen collection (nasal/nasopharyngeal swabs), we surmise that mainly RV and not enterovirus was the pathogen identified. Re-infections were defined by a positive test ≥90 days after the first infection. Viral co-infection was defined by the presence of at least two distinct viruses, using RVP and/or COVID-19/Flu/RSV testing simultaneously or within 14 days of testing.

**Clinical parameters:** The clinical characteristics of children with VRIs were compared. De-identified data abstracted from electronic medical records included demographics, time of infection, clinical findings, immunization status, disease severity, and potential medical comorbidities. All children were classified as having asymptomatic/mild, moderate, or severe acute respiratory illness, using severity classifications categorized by the NIH for COVID-19 [25].

**Comorbidities:** The presence of comorbidities in young children was specifically examined and categorized. Comorbid conditions included chronic lung disease; asthma or reactive airway disease (RAD); prematurity; immunocompromised status due to being on immune suppressants; hematological disorders such as sickle cell disease or malignancies; critically significant congenital heart disease requiring surgical repair; genetic disorders leading to primary immune deficiencies; and neurological diseases such as seizure disorders, cerebral palsy, or other relevant neurological conditions.

**Ethical approvals:** Study activities were approved by the UCLA Institutional Review Board, which provided an IRB exemption.

**Statistical analyses:** Chi-square tests were used to compare basic demographic information between children with detectable respiratory viruses (RSV, RV, FluA/B, CoV, hMPV, adenovirus, and SARS-CoV-2). The primary outcome of the study was severe VRI.

We used logistic regression to evaluate the association between the primary outcome and infection and the respiratory viruses. Logistic regression requires the absence of multicollinearity. To test whether the independent variables were correlated, we calculated Variance Inflation Factors (VIFs), following a previously published guideline that VIF > 5 indicates multicollinearity [26]. Logistic regression was performed with SPSS v.19 (IBM, Inc., Armok, NY, USA).

Odds ratios (ORs) and corresponding 95% confidence intervals (95% CIs) were estimated using the logistic regression with SPSS v.19. The outcome variable was the severity of RV infection, coded as 0 = mild or moderate and 1 = severe or critical. The predictors were coded as follows: (1) race/ethnicity was converted into binary variables using dummy coding; (2) comorbidities were a categorical variable with levels coded as 0 = no comorbidity and 1 = any comorbidity (0 was used as the reference variable); (3) insurance was a binary variable (Medi-Cal was used as the reference variable); sex was a binary variable (male was used as a reference variable); and age was a categorical variable with levels coded as 1 = <1 year, 2 = 1–2 years, and 3 = 3–5 years (3–5 years was used as the reference variable). Adjusted ORs were calculated by including all predictors in the model.

We carried out a subgroup analysis restricted to children infected with RV. The subgroup analysis assessed whether there was an association between the primary outcome and sex, age, race/ethnicity, insurance coverage, and comorbidities.

In all statistical analyses, *p* < 0.05 is considered statistically significant.

## 3. Results

**Figure 1:**
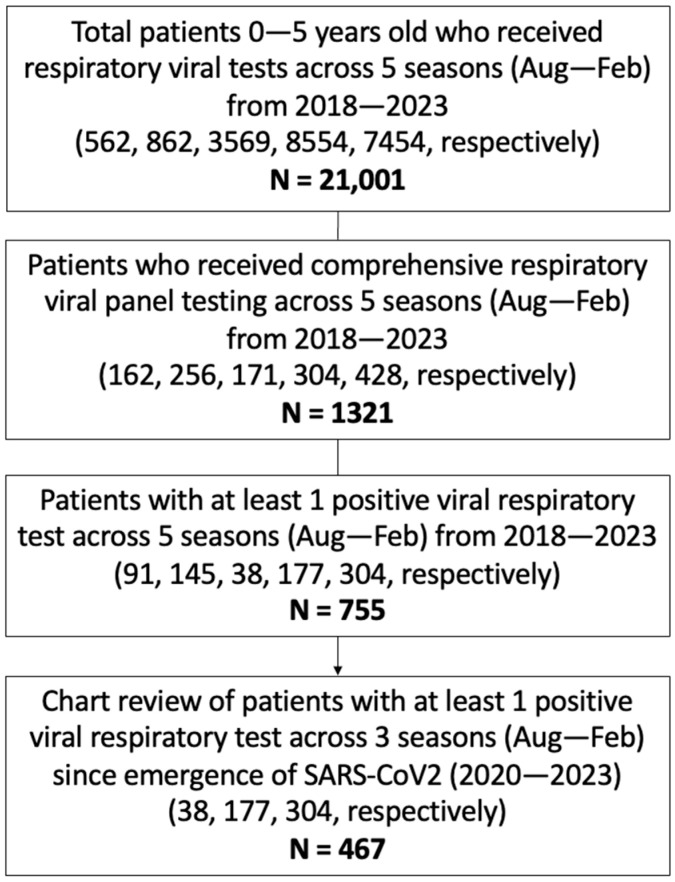
**Study population.** Schematic diagram of inclusion criteria and study cohort selection. cRVP testing includes hMPV, adenovirus, CoV, paraflu 1/2/3/4, RV, RSV, flu A/B, and SARS-CoV2 (since its emergence). Values in parenthesis represent counts of subjects included within the given criterion per season.

In total, 21,001 pediatric patients, aged 0–5 years, received respiratory viral testing through the UCLA Health System across five consecutive respiratory virus seasons (August to February) from 2018 to 2023. A focused retrospective cohort analysis was performed on the 1321 children who underwent cRVP testing, which includes hMPV, adenovirus, CoV, paraflu 1/2/3/4, RV, RSV, flu A/B, and SARS-CoV2 (since its emergence). The study group had a median age of 2 ± 1.53 (SD) years and a female representation of 44%. Among them, 755 patients had at least one detectable viral target in cRVP testing, and from this subgroup, clinical and demographic data were reviewed for the 467 unique patients who had detectable viral infection during the COVID-19 pandemic, constituting the primary focus of the analysis (Figure 1).

**Figure 2:**
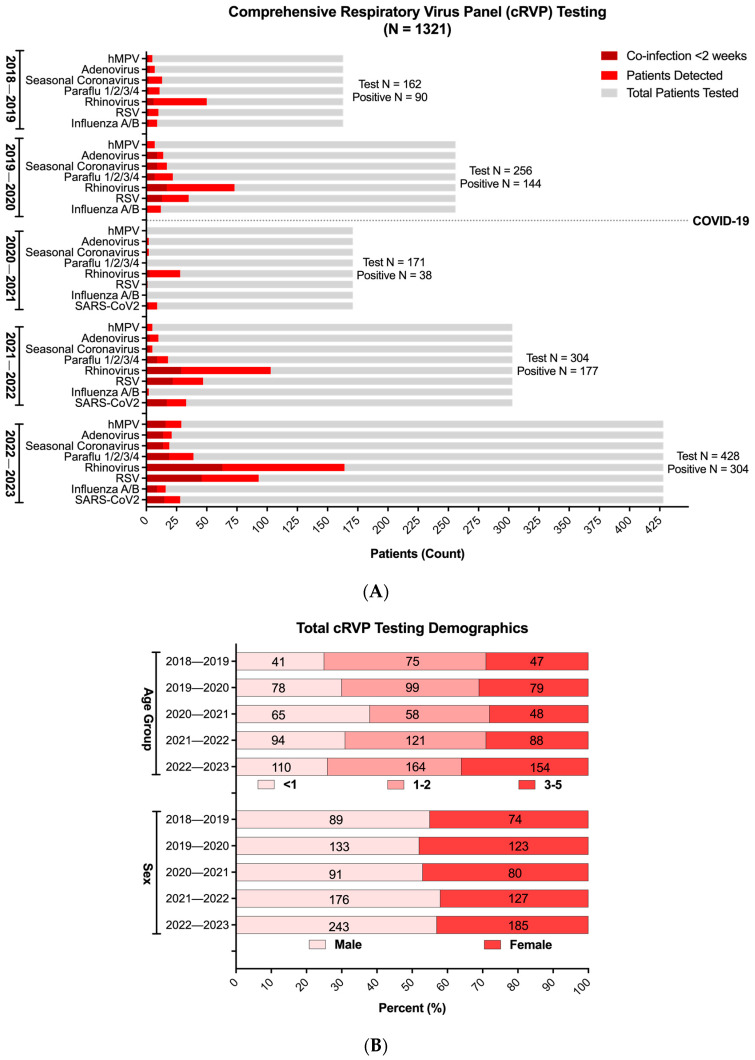
**Comprehensive respiratory virus panel (cRVP) testing in children 0–5 years old (2018–2023).** (**A**) Total cRVP test counts (gray) and respiratory virus positivity (monoviral, red; polyviral, dark red) across five respiratory virus seasons (August to February) from 2018 to 2023. (**B**) Age and sex distribution of patients with at least one positive respiratory virus test result across five respiratory virus seasons (August to February) from 2018 to 2023 (*n* = 755). Age and sex distributions were not significantly different across seasons (*p* > 0.05) using Chi-squared test.

Across the five respiratory seasons, there was an increase in patient testing, starting with an average of 291 patients in the initial two seasons preceding the emergence of SARS-CoV-2, and reaching 428 in the most recent 2022–2023 season (Figure 2A). Proportionally, however, the age and sex distribution remained largely equivalent across the entire study period, except for the immediate COVID-19 season (2020–2021), which favored cRVP testing in the <1-year-old age group (Figure 2B).

With respect to viral positivity, despite the decrease in overall respiratory virus detection during the immediate COVID-19 season, RV, either as a sole pathogen or in conjunction with other viruses, was the predominant respiratory viral infection in the studied pediatric population (Figure 2A, Supplementary Table S1). Although its prevalence among all patients tested exhibited a temporary decline in the 2020–2021 season, to 16% compared to the average 33% observed during the other four seasons, its relative positivity among the patients with VRIs was the highest for the same year, reaching 73.6% (Figure 2A, Supplementary Table S1 and Figure S1A). Following the relaxation of pandemic mitigation and social distancing measures in Los Angeles County in 2021, however, there was a discernible resurgence in the circulation of all the tested respiratory viral illnesses, with RV and RSV re-emerging as the most prevalent viral targets detected (53–58% and 26–30%, respectively, among cRVP-positive patients). By the 2022–2023 season, both viruses surpassed levels observed in the pre-pandemic seasons.

**Figure 3:**
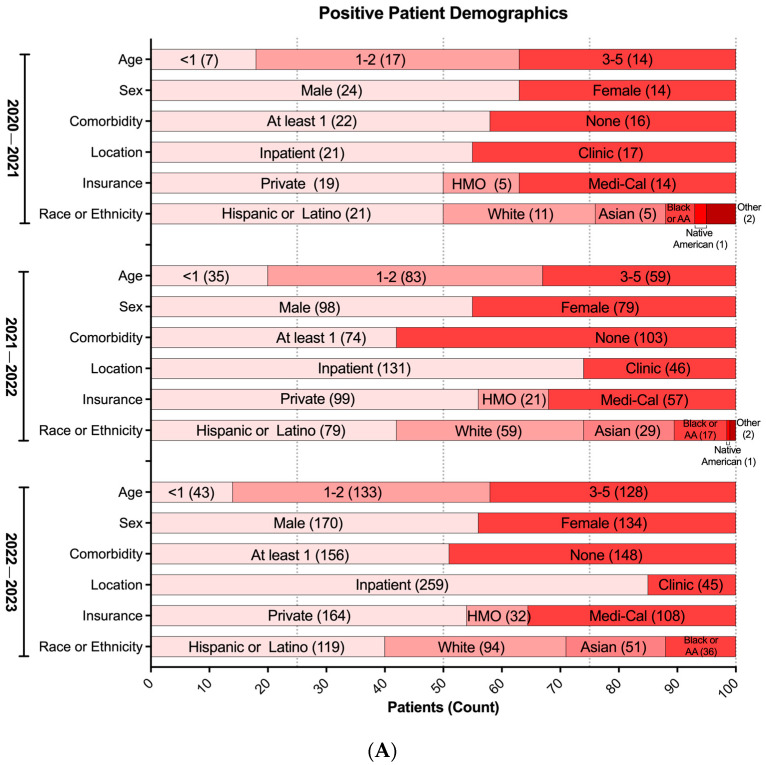

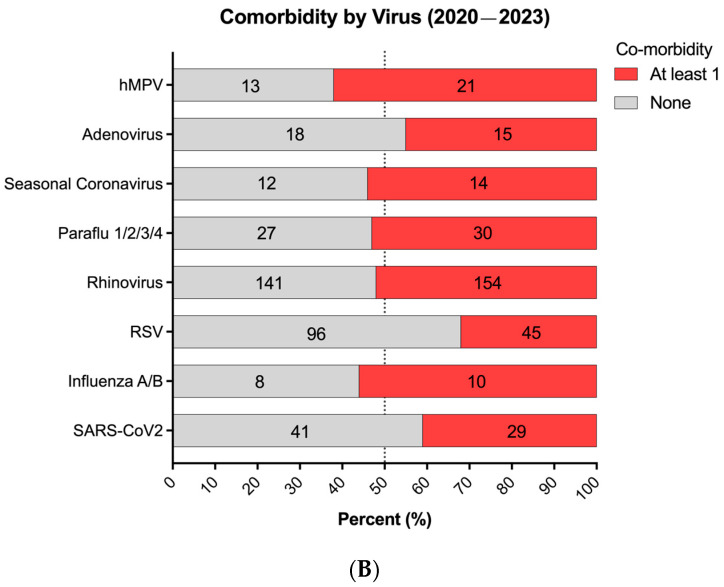
**Respiratory virus positivity during the COVID-19 pandemic (2020–2023).** (**A**) Demographics of patients with at least one positive respiratory virus test from cRVP (*n* = 519). Each demographic variable was not significantly different across seasons (*p* > 0.05) using multinomial logistic regression. HMO: Health Maintenance Organization. (**B**) Proportion of positive patients with or without co-morbidity (*n* = 519). (**A**,**B**) Bars representing the relative proportion. Inset values in bars represent absolute patient counts.

To further describe potential associations between VRIs and multiple parameters within the study cohort during the COVID-19 pandemic, we assessed medical and demographic data from 467 unique patients who tested positive for at least one cRVP target between 2020 and 2023. In Los Angeles County, around 60% of children are of Latino ethnicity [27]; correspondingly, they constitute the largest ethnic group of positive patients collectively across the three seasons. A noticeable shift occurred after the first pandemic year with White, Black or African American, and Asian pediatric groups each showing increases in representation (5% among each ethnic group) with a correlated decrease in the Hispanic or Latino group (10%) (Figure 3A).

We initially observed varying positivity rates between patients with and without comorbidities; although, in the latest season (2022–2023), a balanced distribution emerged, with 49% of overall cases lacking comorbidities and 51% having comorbidities (Figure 3A). Moreover, a composite analysis of all three seasons indicated similar infection rates for each respiratory virus, except for RSV, which notably showed a higher proportion of infected children without pre-existing health conditions compared to those with underlying comorbidities (Figure 3B). Interestingly, the types of comorbidities associated with RSV VRIs were evenly distributed without a discernible predominance, suggesting no significant association with specific underlying health conditions (Figure S1B).

To compare the viral, clinical, and sociodemographic variables over time, we performed a multinomial logistic regression of the children in whom a respiratory virus was detected. In the regression, the dependent variable was a categorical variable representing which virus was detected in the child. The independent variables were the clinical and sociodemographic variables and the respiratory virus season. The results indicated that time, clinical variables, and sociodemographic variables were not significantly associated with viral occurrence (Supplementary Table S2).

**Figure 4:**
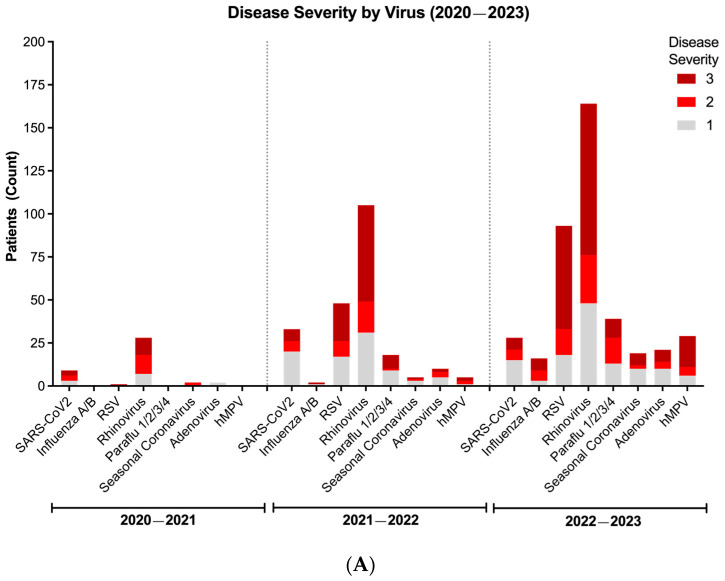

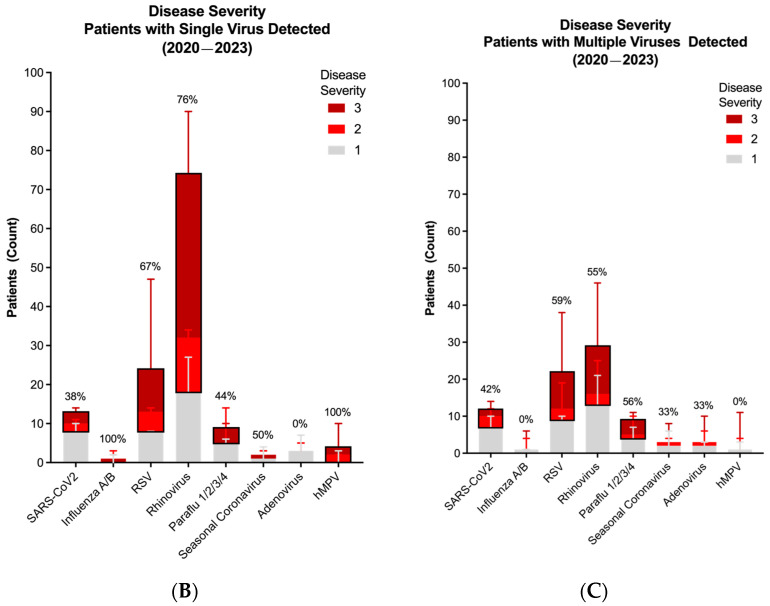
**Disease severity and dynamics of respiratory virus infections during the COVID-19 pandemic (2020–2023).** (**A**) Viral severity according to each virus per season. (**B**) Disease severity of patients with only one positive viral target. (**C**) Disease severity of patients with more than one positive viral target. In Figure (**B**,**C**), bar represents median. Line represents 95% confidence interval. Values represent proportion (%) of patients with moderate-to-severe disease (disease severity score of 2 or 3, black box). Distribution of viral detection was not significantly different across seasons (*p* > 0.05) using Chi-squared test.

To understand trends in disease severity, we utilized NIH’s clinical severity classification system to evaluate the above cohort of patients with at least one positive viral target during the COVID-19 pandemic. Consistent with its high prevalence across seasons, we found that RV consistently led to more cases of moderate-to-severe disease (NIH severity score: 2 or 3), followed by RSV, SARS-CoV-2, and others (Figure 4A). Importantly, five children in our study group died, with two in the 2021–2022 season and three in the 2022–2023 season; three patients were <12 months of age, one 1–2 years of age, and one 3–5 years of age. Four of these deaths were notably associated with RV and the remaining one with SARS-CoV-2.

To identify factors associated with disease severity, we examined viral co-infections, which we defined as the detection of >1 respiratory viral target within the 2-week period since initial testing at the UCLA Health System. Figure 2 shows that the detection of more than one virus in our study cohort was common, contributing 42% of total positive results during the COVID-19 pandemic. Further analysis in co-infections revealed that mirroring total positivity, RV also ranked highest in viral co-infections, followed by RSV, SARS-CoV-2, and others (Figure S2A,B). Not surprisingly, RV most frequently co-infected with the other two most commonly detected targets, RSV and SARS-CoV-2 (Figure S2A,B)

Having established that infection with RV is both the most frequently detected virus, particularly in co-infections, and a significant cause of moderate-to-severe disease, we sought to determine if there was an association between viral dynamics and disease severity. Thus, we compared the frequency of moderate-to-severe disease according to each virus in mono- and polyviral infections. While monoviral RV infections showed a strong association with moderate and severe respiratory illness (NIH severity score: 2 and 3, respectively), this was not significantly different compared to the frequency of moderate-to-severe disease in polyviral infections that included RV (Figure 4B,C). Of note is that the frequency of significant disease with each of the other viruses was also not different whether the virus was present alone or in combination with RV (Figure S2C). Altogether, these data point to RV’s propensity to lead to significant respiratory pathology independently of co-infecting viral pathogens.

**Figure 5:**
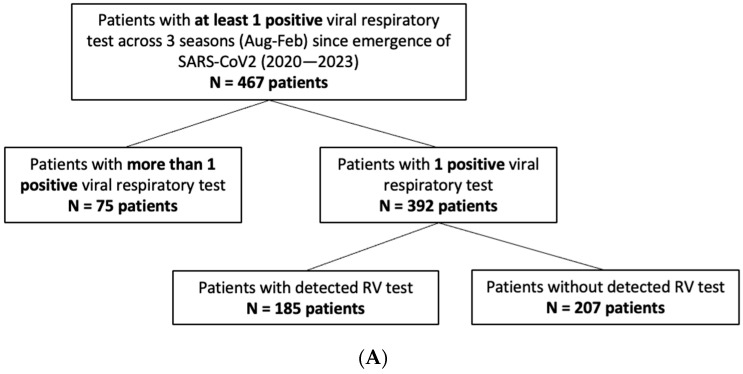

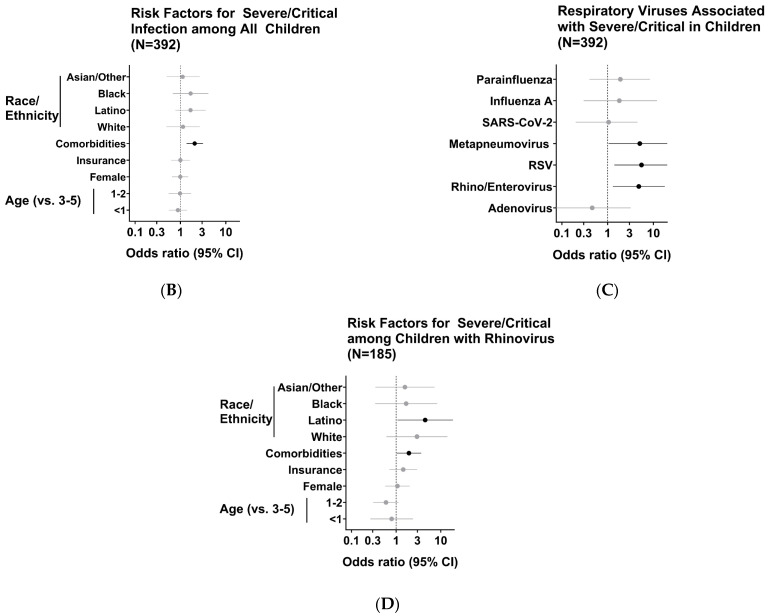
**Identification of viruses and potential associations with development of moderate-to-severe disease.** (**A**) Schematic diagram of inclusion criteria and study cohort selection for risk calculation. Values in parenthesis represent counts of subjects included within the given criterion per season. (**B**) See Table 1; (**C**) see Table 2; (**D**) see Table 3.

Lastly, with the demonstration that RV is the most frequently detected virus in severely infected children, we sought to associate our findings with the risk of critical illness. Using logistic regression analysis, we identified risk factors for the development of moderate-to-severe VRI in our pediatric cohort (Figure 5A). Aligning with the current paradigm of VRIs in young children, pre-existing diagnoses of comorbidities posed a significant risk for the development of serious disease [OR (95% CI): 2.06 (1.36–3.14)] (Figure 5B). Similarly, infections with RSV or hMPV were identified as risk factors for progression to worse disease [OR (95% CI): 5.5 (1.4–21.5), 5.04 (1.05–24.1), respectively]. Most notably, however, infection with RV also emerged as a significant risk factor for serious disease manifestations and hospital admissions (Figure 5C). Additionally, in patients infected with RV, children with comorbid conditions and those identifying within the Hispanic/Latino ethnic group had an added risk for worse outcomes in respiratory pathology [OR (95% CI): 1.94 (1.03–3.65), 4.48 (1.07–18.71), respectively] (Figure 5D). Altogether, our data implicate the emerging role of RV as an important pathogen in a susceptible pediatric population during the COVID-19 pandemic.

## 4. Discussion

The COVID-19 pandemic significantly disrupted viral dynamics, both among hosts and among other viruses. Accordingly, we sought to profile the current landscape of respiratory viral infections in a large metropolitan region to understand disease prevalence and risk factors for severe disease. Our study initially revealed a consistent decline in VRI admissions during the initial phase of the COVID-19 pandemic among young children compared to prior seasons, likely attributed to the altered social practices and preventive measures during the onset of the pandemic [6,7,8]. Subsequently, however, a return in seasonal patterns emerged, marked by an absolute increase in VRIs, aligning with findings from other studies [24,28,29,30,31]. This suggests a normalization of respiratory viral dynamics and epidemiology in a new context of the ongoing COVID-19 pandemic, which prompted our investigations of a vulnerable pediatric population.

Our analysis revealed ethnic disparities in ED visits and hospital admissions among children with VRIs, with Hispanic children experiencing the highest rates of both during the first year of the pandemic. However, in subsequent years, a re-distribution was observed with upticks in VRIs among White, African American, and Asian children, indicating dynamic viral trends across diverse populations influenced by socio-demographic factors and changing community behaviors. Consistent with our study, a separate systematic review and meta-analysis of twenty studies (2016–2022) highlighted racial disparities in VRIs among U.S. children, emphasizing a greater disease burden among Hispanic and African American children, linked to poverty and chronic conditions, and underscoring the importance of vaccinations and other preventative measures for children from these ethnic groups [32].

According to a study by the New Vaccine Surveillance Network (NVSN) conducted between October 2020 and February 2021, RV exhibited comparable or elevated detection rates compared to pre-COVID-19 pandemic months [27] with adjusted odds ratios (aORs) ranging from 1.47 to 3.01 in the ED and from 1.36 to 2.44 in the inpatient setting across all age groups [27]. These findings are consistent with our results and highlight the enduring healthcare burden of these viruses and the need for continuous surveillance in young children, irrespective of the pandemic context. Additionally, it promotes further discussion on the unique characteristics of RV and its resistance to conventional mitigation measures amid a global health crisis. Moreover, despite prior epidemiological studies alluding to the increasing frequency of RV during the COVID-19 pandemic, limited investigations have associated it with severe health outcomes. A recently published single-season study with a smaller cohort of 148 hospitalized pediatric patients (0–5 years) demonstrated that infections with RV, specifically in combination with RSV and/or hMPV, had an impact on treatment length and success. Indeed, two of five patients who were ultimately transferred to the ICU were infected with RV, alone or with co-infection [29].

Consistently in our multi-year cohort study, RV was the primary contributor to moderate-to-severe disease, especially among young children with medical comorbidities. RV, RSV, and hMPV demonstrated significant risks for high disease severity, with RV being particularly notable, leading to increased ED visits and hospital admissions across all age groups. The prevalence of RV and RSV is consistent with a meta-analysis in children of VRIs during the COVID-19 pandemic demonstrating RV as the most frequently detected virus (26.1%), followed by RSV (24.2%) [33]. Interestingly, in our study, hMPV was also identified as a risk factor for severe disease presentation; however, with low detection in our cohort (2.0%), it warrants further investigation in a higher prevalence population to appropriately understand its virulence and clinical impact [33].

More importantly, our study highlights RV’s capacity to cause moderate-to-severe disease independently of co-infection with other viruses. This demonstrates RV’s ability to induce significant pathology in susceptible hosts and alludes to its clinical importance, particularly in pediatric populations. Indeed, despite being conventionally associated with the common cold, recent studies have demonstrated RV’s potential for causing lower respiratory tract infections in children [34]. Initially thought to primarily affect the upper respiratory tract due to better replication at lower temperatures [35], subsequent studies have confirmed its ability to replicate in the higher temperatures of the lower respiratory tract and cause severe respiratory diseases in children, including pneumonia and bronchiolitis [36,37]. In our study, we assessed bacterial co-infection during the COVID-19 seasons and identified limited bacterial–viral co-infection rates. None were detected in 2020–2021 and a frequency of 4% was identified in 2021–2022 (7 patients) and 2022–2023 (13 patients). Pathogens identified included Group A streptococcal infections (four patients), *Haemophilus influenzae* (three patients), *E. coli* (five patients), *Enterobacter cloacae* (two patients), *Klebsiella oxytoca* (two patients), *Enterococcus faecalis*, *Streptococcus anginosus*, *Citrobacter*, *Staphylococcus aureus*, and *Fusarium* spp. (one patient each).

The important findings of our study are balanced with certain limitations that should be acknowledged. With respect to the study population, young children not presenting with symptoms that warrant urgent medical evaluation in an ED or hospital and thus not undergoing cRVP testing were excluded, leading to a narrow estimation of cases. Shifting testing patterns over time, marked by increased access to RT-qPCR testing, may have also influenced case identification, potentially detecting more cases in later periods. Nevertheless, this is unlikely to impact the identification of severe/critical cases, as individuals with more severe symptoms are more likely to seek medical attention and receive cRVP testing. The dynamic nature of testing practices demonstrates an intrinsic limitation of retrospective cross-sectional studies across long time periods due to the ongoing need for adaptable research methodologies to capture the evolving landscape of the pandemic.

Our retrospective chart review also has its own set of limitations. The study’s reliance on data from a specialized academic center may constrain the generalizability of findings to a broader demographic profile. Focusing on children in such a setting introduces a potential bias towards children with multiple comorbidities and medically complex cases. Nevertheless, as the platform for comprehensive viral NAAT is still considerably expensive, it tends to be reserved for more acutely ill children who are at high risk of admission or who are admitted. Once broader availability of comprehensive viral NAAT is attained, additional information on viral dynamics in patients with mild disease will be forthcoming. One additional limitation is that the diagnostic platform used at our institution, the GenMark ePlex Respiratory Viral Panel platform, does not distinguish RV from enteroviruses, although the latter rarely cause respiratory illnesses. Nevertheless, the lack of distinction between RV species such as RVA, RVB, and RVC is a limitation, given that RVC is more frequently associated with lower respiratory infections [30].

In conclusion, our study highlights the high prevalence of RV in young children, even during the COVID-19 pandemic, where it infected more children than SARS-CoV-2. RV independently emerges as a significant contributor to moderate-to-severe respiratory disease, particularly among children with comorbidities, emphasizing the need for targeted interventions and heightened clinical vigilance in vulnerable pediatric populations.

## Figures and Tables

**Table 1:** Odds ratio and 95% confidence interval table according to patient demographics.

Variable	Odds Ratio (95% CI)
Race/ethnicity	
Asian or other	1.12 (0.5–2.66)
Black or African American	1.68 (0.68–4.12)
Hispanic or Latino	1.67 (0.77–3.64)
White	1.14 (0.49–2.68)
Comorbidities	2.06 (1.36–3.14)
Insurance	1 (0.62–1.62)
Female	0.99 (0.65–1.49)
Age (years) *	
1–2	0.98 (0.55–1.73)
<1	0.88 (0.56–1.39)

* The reference group was 3–5 years of age. On each row, the odds ratio describes the risk of severe/critical illness for children of the age in question relative to the reference group.

**Table 2:** Odds Ratio and 95% confidence interval table according to respiratory virus target.

Virus	Odds Ratio (95% CI)
Adenovirus	0.46 (0.07–3.2)
Rhinovirus	4.8 (1.3–17.8)
Respiratory Syncytial Virus	5.5 (1.4–21.5)
Metapneumovirus	5.04 (1.05–24.1)
SARS-CoV-2 virus	1.05 (0.2–4.5)
Influenza A virus	1.8 (0.3–12)
Parainfluenza virus	1.9 (0.4–8.4)

**Table 3:** Odds Ratio and 95% confidence interval table according to patient demographics with detected RV.

Variable	Odds Ratio (95% CI)
Race/ethnicity	
Asian or other	1.58 (0.34–7.270
Black or African American	1.67 (0.34–8.3)
Hispanic or Latino	4.48 (1.07–18.71)
White	2.93 (0.61–14.13)
Comorbidities	1.94 (1.03–3.65)
Insurance	1.44 (0.71–2.96)
Female	1.07 (0.57–2.01)
Age (years) *	
1–2	0.6 (0.31–1.14)
<1	0.8 (0.27–2.39)

* The reference group was 3–5 years of age.

## Data Availability

Data is available upon reasonable request to the corresponding author.

## Supplementary Materials

The following supporting information can be downloaded at https://www.mdpi.com/article/10.3390/idr17020029/s1, Figure S1: Comprehensive respiratory virus testing in children 0–5 years old. A. Age and sex and B. comorbidity distribution of patients with at least one positive respiratory virus test result across five respiratory virus seasons (August to February) from 2018 to 2023; Figure S2: Respiratory virus co-infections during the COVID-19 pandemic (2020–2023). A. Heatmap of co-infections among patients with more than one positive viral target collectively across the COVID-19 pandemic. B. Heatmap of co-infections among patients with more than one positive viral target per season. In Figures A-B Color and number of each cell corresponds to counts of patients with the corresponding viral co-infection combination. Patients with more than two positive results are represented in all binary combinations of viruses. The viruses were ranked in order of total frequency of viral co-infection that includes the corresponding virus. C. Viral disease severity according to each virus co-infecting with rhinovirus. Bar represents median. Line represents 95% confidence interval. Values represent proportion (%) of patients with moderate-to-severe disease (disease severity score of 2 or 3, black box); Table S1: Detection of respiratory virus using the comprehensive respiratory virus panel (cRVP) in children 0-5 years old (2018–2023); Table S2: Comparison of viral, clinical and sociodemographics across seasons.
