# Supplementary material for: Multi-Year Analysis of Respiratory Viral Dynamics Reveals Significance of Rhinovirus in Young Children with Severe Respiratory Illness
Source: Infect Dis Rep. 2025 Apr 3;17(2):29. doi: 10.3390/idr17020029 (PMC12026606; doi:10.3390/idr17020029)
Supplement: Supplementary file 1 [file idr-17-00029-s001.zip › idr-3453741-supplementary.pdf]

SUPPLEMENTARY TABLES:

Supplementary Table S1. Detection of respiratory virus using the comprehensive respiratory virus panel (cRVP) in children 0-5 years old (2018-2023). Total count of each virus detected is noted per season, and further discriminated between counts of single infection and co-infection occurring within 2 weeks initial detection. Values in parenthesis correspond to the relative proportion (%) among the total count of patients with detected virus (n) in each season.

Season	Detection	hMPV	Adenovirus	Seasonal Coronavirus	Paraflu 1/2/3/4	Rhinovirus	RSV	Influenza A/B	SARS-CoV2
**2018-2019 (n=90)**	**Total detected**	**5 (5.5%)**	**7 (7.7%)**	**13 (14.4%)**	**11 (12.2%)**	**50 (55.5%)**	**10 (11.1%)**	**9 (10%)**	**N/A**
	Single Infection	3 (3.3%)	4 (4.4%)	11 (12.2%)	9 (10%)	44 (48.8%)	8 (8.8%)	7 (7.7%)	
	Co-infection <2 weeks	2 (2.2%)	3 (3.3%)	2 (2.2%)	2 (2.2%)	6 (6.6%)	2 (2.2%)	2 (2.2%)	
**2019-2020 (n=144)**	**Total detected**	**7 (4.8%)**	**14 (9.7%)**	**17 (11.8%)**	**22 (15.2%)**	**73 (50.6%)**	**35 (24.3%)**	**12 (8.3%)**	**N/A**
	Single Infection	7 (4.8%)	5 (3.4%)	8 (5.5%)	15 (10.4%)	56 (38.8%)	22 (15.2%)	11 (7.6%)	
	Co-infection <2 weeks	0 (0%)	9 (6.2%)	9 (6.2%)	7 (4.8%)	17 (11.8%)	13 (9%)	1 (0.6%)	
**2020-2021 (n=38)**	**Total detected**	**0 (0%)**	**2 (5.2%)**	**2 (5.2%)**	**0 (0%)**	**28 (73.6%)**	**1 (2.6%)**	**0 (0%)**	**9 (23.6%)**
	Single Infection	0 (0%)	1 (2.6%)	2 (5.2%)	0 (0%)	25 (65.7%)	0 (0%)	0 (0%)	7 (18.4%)
	Co-infection <2 weeks	0 (0%)	1 (2.6%)	0 (0%)	0 (0%)	3 (7.8%)	1 (2.6%)	0 (0%)	2 (5.2%)
**2021-2022 (n=177)**	**Total detected**	**5 (2.8%)**	**10 (5.6%)**	**5 (2.8%)**	**18 (10.1%)**	**103 (58.1%)**	**47 (26.5%)**	**2 (1.1%)**	**33 (18.6%)**
	Single Infection	4 (2.2%)	7 (3.9%)	2 (1.1%)	9 (5%)	74 (41.8%)	25 (14.1%)	1 (0.5%)	16 (9%)
	Co-infection <2 weeks	1 (0.5%)	3 (1.6%)	3 (1.6%)	9 (5%)	29 (16.3%)	22 (12.4%)	1 (0.5%)	17 (9.6%)
**2022-2023 (n=304)**	**Total detected**	**29 (9.5%)**	**21 (6.9%)**	**19 (6.2%)**	**39 (12.8%)**	**164 (53.9%)**	**93 (30.5%)**	**16 (5.2%)**	**28 (9.2%)**
	Single Infection	13 (4.2%)	7 (2.3%)	5 (1.6%)	20 (6.5%)	101 (33.2%)	47 (15.4%)	7 (2.3%)	13 (4.2%)
	Co-infection <2 weeks	16 (5.2%)	14 (4.6%)	14 (4.6%)	19 (6.2%)	63 (20.7%)	46 (15.1%)	9 (2.9%)	15 (4.9%)

Supplementary Table S2. Comparison of viral, clinical and sociodemographics across seasons. Multinomial logistic regression was performed. The dependent variable was a categorical variable representing which virus was detected. The independent variables were the clinical and sociodemographic variables, and the respiratory virus season.

Variable	Chi-squared	p
**Comorbidities**	4.37	0.224
**Insurance**	1.2	0.753
**Female sex**	1.97	0.579
**Time**	4.43	0.619
**Age**	4.4	0.221

SUPPLEMENTARY FIGURES:

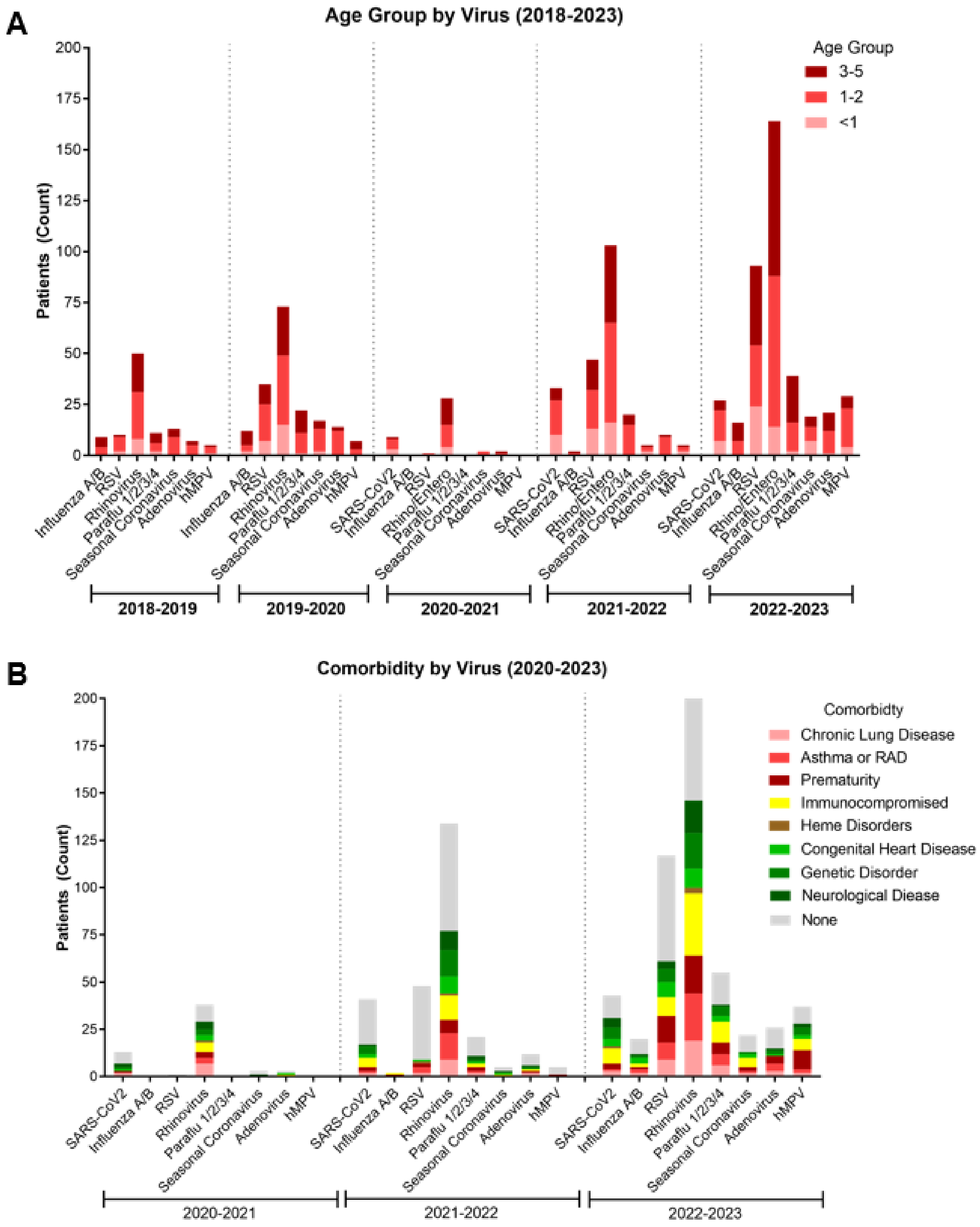

Figure S1. Comprehensive respiratory virus testing in children 0-5 years old. A. Age and sex and **B.** comorbidity distribution of patients with at least one positive respiratory virus test result across five respiratory virus seasons (August to February) from 2018 to 2023.

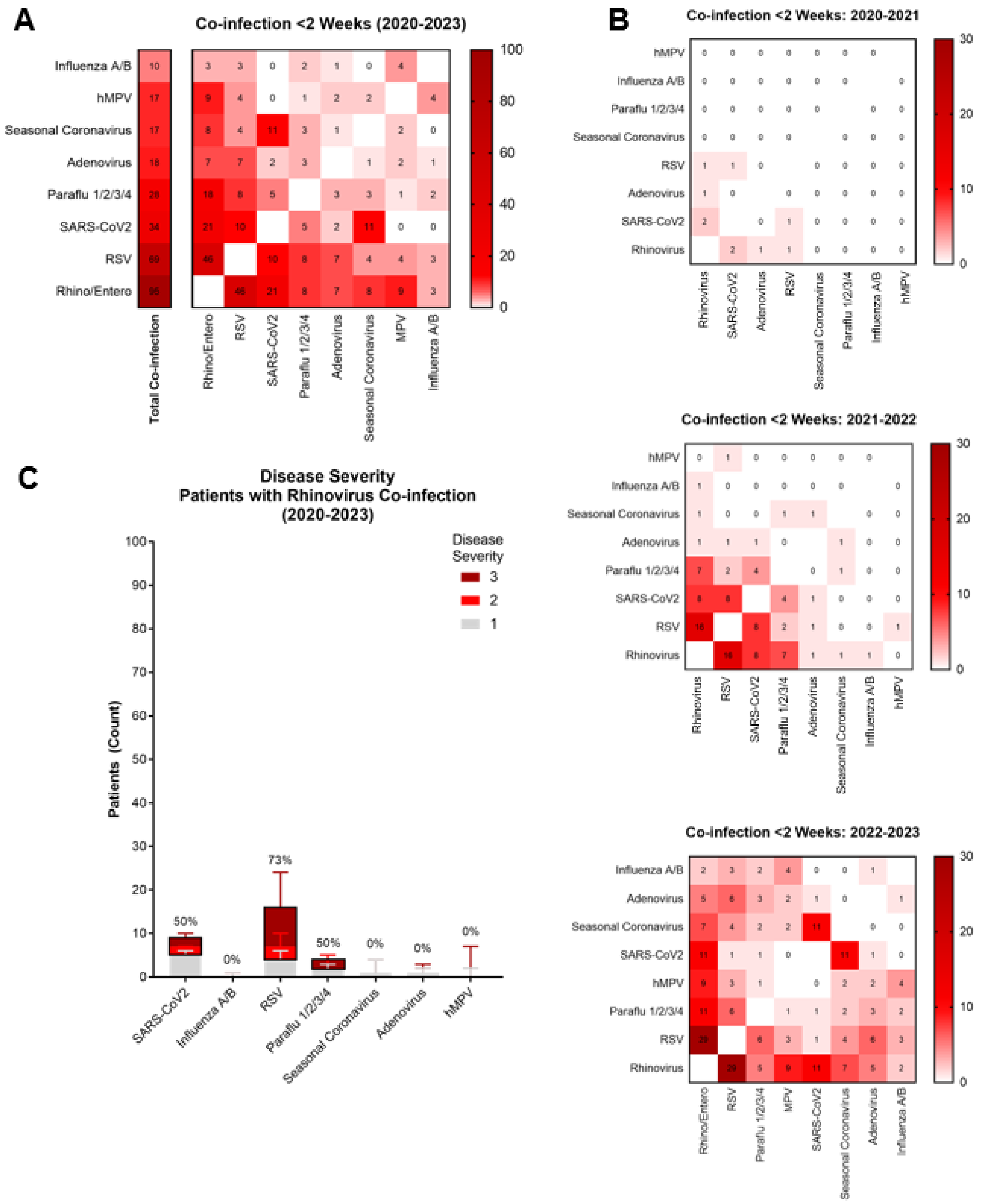

Figure S2. Respiratory virus co-infections during the COVID-19 pandemic (2020-2023). A. Heatmap of co-infections among patients with more than one positive viral target collectively across the COVID-19 pandemic. **B.** Heatmap of co-infections among patients with more than one positive viral target per season. In Figures **A-B** Color and number of each cell corresponds to counts of patients with the corresponding viral co-infection combination. Patients with more than two positive results are represented in all binary combinations of viruses. The viruses were ranked in order of total frequency of viral co-infection that includes the corresponding virus. **C.** Viral disease severity according to each virus co-infecting with rhinovirus. Bar represents median. Line represents 95% confidence interval. Values represent proportion (%) of patients with moderate-to-severe disease (disease severity score of 2 or 3, black box).
